# Supplementary material for: The causal role of circulating inflammatory markers in osteoporosis: a bidirectional Mendelian randomized study
Source: Front Immunol. 2024 Jul 18;15:1412298. doi: 10.3389/fimmu.2024.1412298 (PMC11291241; doi:10.3389/fimmu.2024.1412298)
Supplement: Supplementary file 1 [file DataSheet_1.zip › supplementary material/CIM-OSTEOPOROSIS /GCST90274837.csv_leave_one_out.pdf]

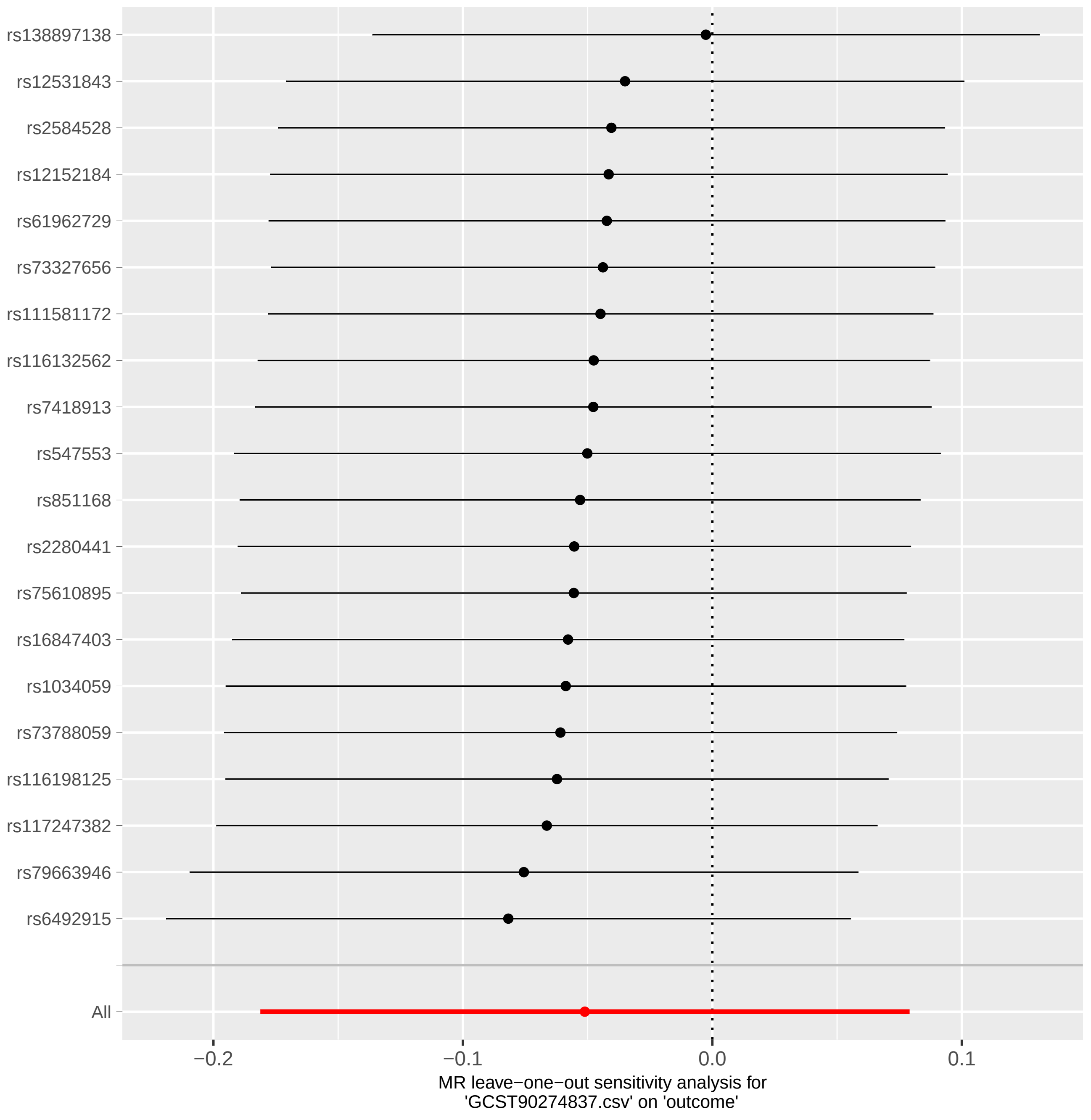
rs138897138
rs12531843
rs2584528
rs12152184
rs61962729
rs73327656
rs111581172
rs116132562
rs7418913
rs547553
rs851168
rs2280441
rs75610895
rs16847403
rs1034059
rs73788059
rs116198125
rs117247382
rs79663946
rs6492915
All
−0.2
−0.1
0.0
0.1
MR leave−one−out sensitivity analysis for
'GCST90274837.csv' on 'outcome'
